# CRISPR–Cas9-mediated functional dissection of 3′-UTRs

**DOI:** 10.1093/nar/gkx675

**Published:** 2017-08-03

**Authors:** Wenxue Zhao, David Siegel, Anne Biton, Olivier Le Tonqueze, Noah Zaitlen, Nadav Ahituv, David J. Erle

**Affiliations:** 1 Lung Biology Center, Department of Medicine, University of California San Francisco, 4th St, San Francisco, CA 94158, USA; 2 Centre de Bioinformatique, Biostatistique et Biologie Intégrative, C3BI, USR 3756 Institut Pasteur et CNRS, 25–28 Rue du Dr Roux, Paris 75015, France; 3 Department of Bioengineering and Therapeutic Sciences, Institute for Human Genetics, University of California San Francisco, 4th St, San Francisco, CA 94158, USA

## Abstract

Many studies using reporter assays have demonstrated that 3′ untranslated regions (3′-UTRs) regulate gene expression by controlling mRNA stability and translation. Due to intrinsic limitations of heterologous reporter assays, we sought to develop a gene editing approach to investigate the regulatory activity of 3′-UTRs in their native context. We initially used dual-CRISPR (clustered, regularly interspaced, short palindromic repeats)-Cas9 targeting to delete DNA regions corresponding to nine chemokine 3′-UTRs that destabilized mRNA in a reporter assay. Targeting six chemokine 3′-UTRs increased chemokine mRNA levels as expected. However, targeting *CXCL1, CXCL6* and *CXCL8* 3′-UTRs unexpectedly led to substantial mRNA decreases. Metabolic labeling assays showed that targeting these three 3′-UTRs increased mRNA stability, as predicted by the reporter assay, while also markedly decreasing transcription, demonstrating an unexpected role for 3′-UTR sequences in transcriptional regulation. We further show that CRISPR–Cas9 targeting of specific 3′-UTR elements can be used for modulating gene expression and for highly parallel localization of active 3′-UTR elements in the native context. Our work demonstrates the duality and complexity of 3′-UTR sequences in regulation of gene expression and provides a useful approach for modulating gene expression and for functional annotation of 3′-UTRs in the native context.

## INTRODUCTION

3′ untranslated regions (3′-UTRs) have extensive effects on gene expression during development (1), aging (2), inflammation (3) and in diseases including cancer (4). These processes are precisely controlled by many types of *cis*-regulatory elements such as AU-rich elements (AREs) (5), Pumilio motifs (5), constitutive decay elements (6) and microRNA targeting sites (7), which modulate mRNA stability and/or translation through binding to RNA binding proteins or microRNAs (8). For example, AREs, which consist of one or more of UAUUUAU repeats, can either increase or decrease gene expression. For instance, the ARE binding protein TTP can regulate tumor growth through interacting with the 3′-UTRs of tumor necrosis factor (*TNF-α*), cyclooxygenase 2 (*COX-2*) and vascular endothelial growth factor (*VEGF*) (4,9). MicroRNAs are conserved small RNAs that regulate protein translation and mRNA decay by binding to the 3′-UTRs of target mRNAs and thereby inhibit gene expression. It is estimated that more than 60% of the human protein-coding genes harbor miRNA target sites in their 3′-UTRs (1). The interplay between microRNAs and 3′-UTRs affects almost all known biological processes, including cell growth, proliferation and differentiation, as well as development and disease (1,10).

Evaluation of 3′-UTR activity typically relies upon the use of heterologous reporter systems in which 3′-UTRs are placed downstream of the stop codon of a reporter transgene (11,12). Reporter assays are convenient and can be used for massively parallel analyses of large sets of 3′-UTR sequences (11,12). However, since reporters cannot detect 3′-UTR effects in their native context, these assays fail to account for potential interactions between the 3′-UTR and other sequences, including 5′-UTRs, protein-coding regions and introns. We therefore sought to develop an approach that could be used to evaluate the effects of 3′-UTRs on gene expression in the native context. Cas9, an RNA-directed DNA endonuclease, is a powerful tool that enables CRISPR (clustered, regularly interspaced, short palindromic repeats)-guided genome manipulation (13,14). In this work, we have used CRISPR/Cas9 to target genomic DNA transcribed into 3′-UTRs (which we refer to as ‘3′-UTR DNAs’), which allowed us to observe the effects of loss of 3′-UTRs in the genomic context and compare these effects with the effects seen in reporter assays. A 4sU (4-thiouridine, a uridine analog) metabolic labeling assay was used to monitor the effects of targeting 3′-UTRs on dynamics of transcripts in the native context. We found that targeting certain 3′-UTRs can affect both gene transcription and mRNA decay. Furthermore, we demonstrated that CRISPR–Cas9 targeting of 3′-UTR *cis*-elements could be used to modulate gene expression. We also developed a highly parallel approach based on a guide RNA (gRNA) library for localizing specific 3′-UTR elements in their native context.

## MATERIALS AND METHODS

### 3′-UTR reporter construction

The 3′-UTR reporter BTV has been described previously (11). BTV contains an *LNGFR* reference gene and a green fluorescent protein (GFP) reporter gene, both driven by a bi-directional tetracycline-regulated promoter. 3′-UTR sequences were amplified from human genomic DNA (G304A, Promega) using a forward primer containing an MluI site and a reverse primer containing a PacI site. Both the 3′-UTR polymerase chain reaction (PCR) product and BTV were digested with MluI and PacI and the products were ligated together. Reporter lentiviruses were produced as described previously (11).

### 3′-UTR reporter assays

BEAS-2B human bronchial epithelial cells stably transduced with a tetracycline transactivator transgene (Beas2B.tTA cells) were cultured in Dulbecco's modified Eagle’s medium (DMEM) (high glucose) with 10% fetal bovine serum (FBS), glutamine and nonessential amino acid at 37°C, 5% CO_2_ and 100% humidity. For transduction, conditioned medium containing lentivirus and fresh medium (1:3) with polybrene (final concentration 8 μg/ml) was added to the cells. The medium was replaced with fresh medium 24 h after adding virus and cultured for at least 2 weeks. For analysis of steady state reporter (GFP) mRNA level, the cells were harvested by adding Buffer RLT (Qiagen) followed by isolation of RNA and genomic DNA. RNA was reverse transcribed into complementary DNA (cDNA) using the SuperScript^®^ III First-Strand Synthesis System (Invitrogen). The Power SYBR Green PCR Master Mix (Thermo, 4367659) was used for quantitative real-time PCR (qRT-PCR). *GFP* mRNA levels were normalized using *LNGFR* as a housekeeping gene. Effects of chemokine 3′-UTRs on steady state mRNA levels were calculated as (normalized *GFP* mRNA for reporter with chemokine 3′-UTR)/(normalized *GFP* mRNA for empty reporter). To analyze mRNA decay, Beas2B.tTA cells were treated with doxycycline (Dox) (1 μg/ml) and harvested after 0 and 4 h (t_0_ and t_4_) by adding Buffer RLT (Qiagen). Total RNA was extracted using the RNeasy mini kit (Qiagen) and reverse transcribed into cDNA using an oligo-dT primer. GFP mRNA levels were quantified with qRT-PCR and normalized to the house keeping gene *GAPDH*. The remaining mRNA after 4 h was normalized to the t_0_ mRNA (t_4_/t_0_) and was used to determine mRNA stability.

### pLX-dual-gRNA and pCW-Csy4-hygromycin plasmid construction

The pLX-dual-gRNA and pCW-Csy4-hygromycin lentiviral plasmids were produced for expression of pairs of gRNAs. PCR primer and other oligonucleotide sequences used in this work are shown in Supplementary Data. PCR was performed using Phusion High-Fidelity PCR master mix with HF buffer (NEB, M0531S) at 98°C for 30 s; followed by 28 cycles of 98°C for 30 s, 65°C for 30 s and 72°C for 30 s; followed by a final incubation at 72°C for 5 min. Ligations were performed using T4 DNA ligase (NEB). *Mix & Go* Competent Cells—Strain Zymo 5α (Zymo Research, T3007) were used for all transformations in this work and all plasmids were verified by sequencing.

The pLX-dual-gRNA lentiviral vector was used to drive expression of a single transcript containing two gRNAs separated by a Csy4 cleavage site. pLX-dual-gRNA was derived from pLX-sgRNA-AAVS1 (Addgene #50662) (15) and pSQ1313 (Addgene #53370) (16). Two fragments of pSQT1313 were amplified using the primer pair pLX-*Nde*I-F1 and pLX-R (PCR1), and the primer pair pLX-F2 and pLX-*Nhe*I-R2 (PCR2). Equal volumes of PCR1 and PCR2 products were used as templates for a second-round PCR with the primers pLX-*Nde*I-F1 and pLX-*Nhe*I-R2 to produce the product PCR3. PCR3 and pLX-sgRNA-AAVS1 were each digested with *Nde*I and *Nhe*I and the digestion products were ligated to produce pLX-dual-gRNA. To insert pairs of gRNAs (gRNA_1 and gRNA_2) into this construct, we performed a single 60 μl PCR with five oligonucleotides (3 μl of each): Mid 90 (0.1 μM), sgRNA_1 (0.1 μM), sgRNA_2 (0.1 μM), attachment1 (10 μM) and attachment2 (10 μM). The PCR product and pLX-dual-gRNA were digested with *Bsp*MI prior to ligation to produce the final dual gRNA constructs.

The pCW-Csy4-Hygromycin lentiviral vector was produced as a means to express the ribonuclease Csy4, which was used to cleave Csy4 sites in pLX-dual-gRNA transcripts and produce a pair of gRNAs. To make pCW-Csy4-Hygromycin, a fragment of pGL4.11 (Addgene #59744) was amplified using primers Hyg-F and Hyg-XbaI-R (PCR4) and a fragment of pCW-Cas9 (Addgene #50661)^2^ was amplified using primers hPGK-SpeI-F and hPGK-R (PCR5). Equal volumes of PCR4 and PCR5 were used as templates for a second-round PCR with the primers hPGK-SpeI-F and Hyg-XbaI-R (PCR6). PCR6 and pCW-Cas9 were digested with SpeI and XbaI and ligated to produce pCW-Csy4-Cas9. To add a hygromycin resistance gene, we replaced Cas9 with hygromycin as follows. The hygromycin gene from pSQT834 (Addgene# 53371) (16) was amplified using the primers Csy4-Nhe-F and Csy4-BamHI-R (PCR7). The PCR7 product and pCW-Cas9-hygromycin were digested first with *Nhe*I and then with BamHI and the products were ligated together to generate pCW-Csy4-Hygromycin.

### CRISPR targeting of 3′-UTRs

To efficiently excise, the majority of each 3′-UTR sequence, we used a construct expressing a precursor RNA that was processed by Csy4 to deliver two gRNAs. One gRNA targeted a sequence in the region following the stop codon and the other targeted a sequence in the region preceding the polyA signal sequence. In cases where the canonical polyA signal sequence AAUAAA was not present between 10 and 35 nt upstream of the polyA site, we used PolyAPred (17) to identify the polyA signal. To avoid deleting any portion of the coding sequence or stop codon, proximal gRNAs were based on the antisense strand with the NGG protospacer adjacent motif (PAM) located within the proximal 3′-UTR. To avoid deleting the polyA signal sequence, distal gRNAs were based on the sense strand with the NGG PAM located upstream of the polyA signal. Positions of each of these paired gRNAs relative to stop codons and polyA sites are shown in Supplementary Data. For dual CRISPR targeting experiments, BEAS-2B.tTA cells were transduced with pLX-dual-gRNA (which contains a blasticidin resistance gene), pCW-Csy4-hygromycin and LentiCRISPRv2 (which contains a Cas9 gene and a puromycin-resistance gene, Addgene #52961) (18). Triply transduced cells were selected in medium containing blasticidin, hygromycin and puromycin for at least 3 weeks prior to DNA and RNA extraction (All Prep DNA/RNA mini kit, Qiagen). All studies were performed using polyclonal populations; sample sizes refer to the number of technical replicates performed with each polyclonal population. To detect the 3′-UTR deletions, the DNA was PCR amplified with a pair of primers flanking the two sgRNA target sites and analyzed by agarose gel electrophoresis. Chemokine mRNA levels were measured by qRT-PCR and normalized using *GAPDH* mRNA.

### Measurement of mRNA decay and transcription using 4sU metabolic labeling

Beas2B.tTA cells were plated in 15 cm dishes with 30 ml medium 1 day before labeling and were ∼80% confluent when 4sU was added. To prepare a 40 mM 4sU stock solution, 100 mg of 4sU (Sigma, T4509) was dissolved in 9.6 ml phosphate buffered saline (PBS) and used immediately. To monitor mRNA decay (19), 300 μl of 4sU stock solution was added to each plate and mixed into the medium by gentle shaking followed by a 4 h incubation. After washing cells twice with 25 ml PBS (pre-warmed to 37°C), cells were harvested immediately (t_0_) or maintained in an incubator for 4 h (t_4_). To measure newly synthesized transcripts, 375 μl of 4sU stock solution was added to each plate; after a 20 min incubation cells were washed with 20 ml ice-cold PBS and then harvested in Buffer RLT for total RNA extraction with the RNeasy mini kit.

The 4sU labeled RNAs in the total RNA isolates were biotinylated with the following procedure. A 50 mg EZ-Link Biotin-HPDP (Pierce, 21341) was dissolved in 50 ml of dimethylformamide (DMF) to produce 1 mg/ml biotin stock solution. The labeling reaction was carried out in siliconized 15 ml tubes containing 300 μl 10× TE buffer (100 mM Tris pH7.4 + 10 mM ethylenediaminetetraacetic acid (EDTA)), 600 μl biotin stock solution, 300–400 μg total RNA and water to 3 ml. The tubes were kept in the dark and rotated for 2 h. To remove free chemicals, an equal volume (3 ml) of chloroform/isoamylalcohol (24:1) was added to the reaction and hand shaken vigorously to homogenize the mixture. After transfer to MaXtract high density tubes (Qiagen), hand shaking to produce a homogenous suspension, waiting for 2–3 min and then spinning at 1500 *g* for 6 min at 4°C, the upper aqueous phase was transferred to multiple 1.7 ml tubes. After addition of 1/10 volume of 5 M NaCl and one volume of isopropanol, tubes were inverted to mix thoroughly and incubated at room temperature for 10 min and at −20°C for 20 min. After spinning at 20 000 × *g* for 30 min at 4°C, removing the supernatant, adding one volume of 75% ethanol and spinning at 20 000 × *g* for 15 min at 4°C, the supernatant was removed and briefly air-dried. Pellets were re-dissolved in 50 μl water, combined and stored at −80°C.

Biotin labeled 4sU RNA was isolated with the following procedure. A total of 300 μg total RNA containing biotinlylated RNAs was mixed with 10× TE buffer and water to produce a final RNA concentration of ∼100 μg/μl, followed by heating at 65°C for 10 min and immediately placing tubes on ice for 5 min. Next, we used μMACS streptavidin beads and columns (Miltenyi, 130–074-101) to isolate 4sU RNAs according to the manufacturer’s instructions. Final 4sU RNA elutes were purified with RNeasy MinElute Spin columns (Qiagen, 74204) in RNase free water. The resulting RNAs were reverse transcribed into cDNA with oligo-dT primer and used for qRT-PCR. GAPDH mRNA was used for normalization. We quantified chemokine mRNA stability by determining the relative fraction of RNA remaining after 4 h: ([chemokine RNA at 4 h]/[*GAPDH* at 4 h])/([chemokine RNA at 0 h]/[*GAPDH* at 0 h]). We quantified newly synthesized chemokine mRNA as the ratio of ([chemokine RNA after 20 min pulse]/[*GAPDH* RNA after 20 min pulse])/([mean wild-type chemokine RNA after 20 min pulse]/[mean wild-type *GAPDH* RNA after 20 min pulse]).

### Enhancer–reporter assay

The enhancer–reporter (pLS-mp) contains an enhanced green fluorescent protein (EGFP) transgene, a minimal promoter and cloning sites for insertion of candidate enhancer sequences (20). To clone 3′-UTR DNA sequences into pLS-mP, sequences were amplified with gene specific primers containing XbaI and SbfI sites. The PCR product and the plasmid were each digested with XbaI and SbfI and ligated together to produce reporter plasmids. These plasmids were packaged in lentivirus and used to transduce Beas2B.tTA cells. To analyze reporter expression, genomic DNA and total RNA were extracted from the transduced cells, followed by reverse transcription of RNA into cDNA and analysis with qRT-PCR. *GFP* mRNA and DNA levels were normalized using *GAPDH* as a reference gene and enhancer activity was calculated as (normalized GFP mRNA level)/(normalized GFP DNA level).

### Highly parallel analysis of 3′-UTR deletions

For a pilot analysis, we designed a small set of gRNAs targeting six sites in and around the N1N2 element in the 3′-UTR of *CXCL3* mRNA. Oligonucleotides containing these single gRNA sequences (Supplementary Data) were cloned into the plasmid MP283_mCD4_Cas9, which was modified from pSicoR by transferring Cas9 expression cassette and sgRNA cloning sites into it. Equal amounts of the six plasmids were used to transfect Beas2B.tTA cells in 10-cm dishes (∼80% confluent). After selection with puromycin and 2 weeks in culture, cells were harvested for DNA and total RNA extraction. Total RNA was reverse transcribed into cDNA and genomic DNA and cDNA was amplified by PCR using primers flanking the target sites (Supplementary Data). PCR was performed by incubating at 98°C for 45 s; followed by 15 cycles of 98°C for 15 s, 60°C for 30 s and 72°C for 60 s, and a final incubation at 72°C for 60 s, using Kapa HiFi polymerase (Kapa Biosystem, KK2611). The first-round PCR product was used for a second-round PCR with the primer pair D2nd-F and D2nd-R (Supplementary Data) (98°C for 45 s; followed by 18 cycles of 98°C for 15 s, 60°C for 30 s and 72°C for 60; and 72°C for 60 s; Kapa HiFi polymerase). The PCR product was gel purified and pooled for sequencing on Illumina Hiseq 2500. We identified the nine most abundant deletions of each of three types: (i) deletions within the *CXCL3* N1N2 element (**Δ** N1N2); (ii) deletions that excised the entire N1N2 element and some flanking sequence (**Δ** N1N2+) and (iii) control deletions that did not involve the N1N2 element (**Δ** C). For each deletion, we determined the ratio of read counts from the RNA sample to read counts for the DNA sample and normalized this to the ratio of *CXCL3* 3′-UTR read counts without deletions. Ratios for the **Δ** N1N2 and **Δ** N1N2+ deletions were compared with **Δ** C deletions using the Mann–Whitney U-test.

For testing a complete set of oligonucleotides targeting the *CXCL1* 3′-UTR, a pool of oligonucleotides corresponding to all possible gRNA recognition sites (Supplementary Data) was synthesized by Custom Array. The pool was amplified by PCR using the forward primer IVT.lib_CXCL1 and the reverse primer IVT.lib_R (Supplementary Data). The PCR product was gel purified and used as the template for *in vitro* transcription of sgRNAs (MEGAshortscript Kit, Thermo, AM1354). The sgRNA transcripts were purified with MEGAclear Kit (Thermo, AM1908). A total of 100 ng total of the sgRNA pool was mixed with 500 ng Cas9 protein (PNA Bio, CP01) and used to transfect 50 000 Beas2B.tTA cells in a 24-well plate well with Lipofectamine RNAiMAX (Thermo, 13778030). The medium was changed 16 h post-transfection. The transfected cells were propagated and passaged for 2 weeks; by the time of harvesting the cells for DNA and RNA extraction, the cells were ∼70–80% confluent in a 15-cm dish. All of the isolated RNA was used to prepare cDNA. To make the RNA and DNA sequencing libraries, all of the cDNA and genomic DNA was amplified by PCR with primers CXCL1.P1_F20 and CXCL1.P3.R30 (Supplementary Data). The PCR was performed with TaKaRa Ex Taq polymerase (Clontech, RR001A) and limited to 15 cycles. The unpurified PCR product was used as template for a second round of PCR with Kapa HiFi PCR master mix (Kapa Biosystem, KK2611) and the primers CXCL1full_Nx_F and CXCL1full_Nx_R (Supplementary Data). PCR products were gel purified and used as templates to prepare the sequencing libraries using the Nextera XT kit (Illumina, 15052164). The libraries were then sequenced using an Illumina HiSeq 2500 (150 nt paired end reads, ∼80 M DNA reads and 45 M cDNA reads).

### Analysis of the high-throughput CRISPR assay data

Reads were aligned to the reference sequence using STAR aligner (21), with default settings in genome generate mode and genome SA index N bases = 4. For mapping mode we used end-to-end read alignment, maximum of 20 mismatches, minimum of 40 matched bases and disallowed multiple alignments. FastQC was used to check read quality. Samtools sort was used to sort by read name for paired-end reads. After mapping, we wrote custom scripts to ignore the following: any reads with a deletion <8 nt from the start or end of the read, since uncertainty in that region can be high; any deletions of length 1 or 2 bases, since past experience has shown that these are often likely to be sequencing errors; any reads with multiple deletions; and any deletions 30 nt or longer. Unmapped regions of the 3′-UTR were assumed to have no deletions. If no deletions were detected, the read corresponded to the reference sequence.

We grouped any number of reads of DNA molecules with the same deletion to be considered a single ‘distinct deletion’. After sorting counts into distinct deletions, we further eliminated from our analysis any distinct deletion with ≤6.5 reads/10^7^ reads (8 total reads of RNA or 16 total reads of DNA). We calculated the count ratio: *r* = (RNA counts)/(DNA counts).

For each position along with the 3′-UTR, we identified the set of deletions that involved that position and calculated the median value, and 95% confidence interval for *r* by following a standard bootstrap algorithm. To estimate *P*-values for each position, we used a standard Mann–Whitney U-test to compare the distribution of *r* at a given location to the distribution of *r* obtained from all other distinct deletions. The total *r* for all distinct deletions was 0.47, which was similar to the reference sequence ratio of 0.51. Positions with fewer than four distinct deletions were not included in the analysis.

### Data analysis and statistics

Data are displayed as mean ± s.d., with sample size indicated in figure legends. No randomization or blinding was performed. Sample size estimation was performed for the starting number of cells in the high-throughput experiment by considering both read depth and indel coverage; any sequences with deletions <3 or >29 nts were excluded from analysis. Statistical analysis was performed using two-sided Student’s *t*-test, one-way ANOVA and Dunnett’s test, or Mann–Whitney U-test as indicated in the legends.

### Illumina sequencing data

The high-throughput sequencing data are stored at SRA (SRP100490, https://www.ncbi.nlm.nih.gov/Traces/study/?acc=SRP100490).

## RESULTS

### 3′-UTRs from nine chemokines inhibited reporter gene expression

**Figure 1.**
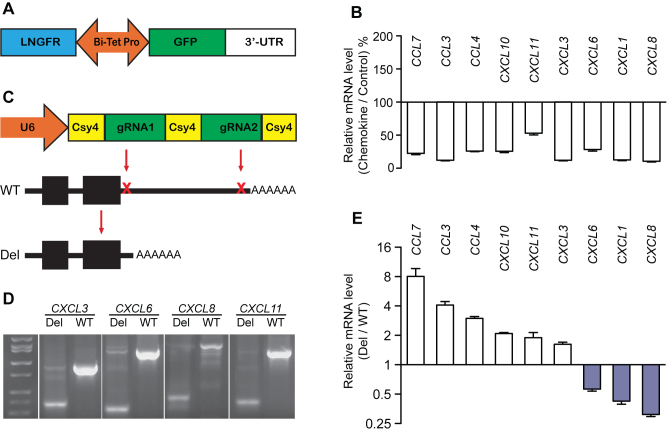
Comparison of the effect of chemokine 3′-UTRs on steady state mRNA levels in the reporter and native contexts. (**A**) The reporter construct (BTV), which contains a bi-directional promoter that drives both GFP and LNGFR expression. This promoter is under control of Tet-Responsive Element; transcriptional activity can be terminated by doxycycline (Dox). The 3′-UTR is cloned downstream of the GFP stop codon. GFP expression is normalized using the LNGFR reference transgene and the effect of 3′-UTR is quantified as the ratio GFP/LNGFR. (**B**) Effects of nine chemokine 3′-UTRs on reporter GFP mRNA levels, *n* = 3. (**C**) The dual-CRISPR (clustered, regularly interspaced, short palindromic repeats) system for deleting 3′-UTRs in the genomic context. U6 promoter drives expression of a transcript containing two guide RNAs (gRNAs) that are excised by co-expressed Csy4 nuclease. The resulting two gRNAs cleave sites in the proximal and distal 3′-UTR. (**D**) PCR verification for deletion of chemokine 3′-UTR DNAs in the genome. Polymerase chain reaction (PCR) was performed with DNA isolated from polyclonal populations of dual-CRISPR edited cells by using primers flanking the two gRNA sites. A successful deletion resulted in a smaller PCR amplicon. Four examples are shown. (**E**) Effects of targeting nine chemokine 3′-UTRs in the native context on mRNA levels, *n* = 5 technical replicates from each polyclonal population. All values represent mean ± s.d., all *P-*values < 0.05 by two-sided Student’s *t*-test.

Chemokines are a family of chemotactic cytokines which play crucial roles in cell migration and are important in immune responses, the nervous system and cancer (22). Previous reporter assays have indicated that 3′-UTRs of many chemokines, including *CXCL1*, CXCL3 and *CXCL8* (*IL-8*) (11), are highly active in post-transcriptional gene regulation. We cloned 3′-UTRs from nine human chemokine mRNAs into a 3′-UTR reporter (BTV) (Figure 1A). We used qRT-PCR to measure the effects of these 3′-UTRs on steady state level of the GFP reporter mRNA. Compared with the control (BTV empty, no 3′-UTR test sequence), each of the nine 3′-UTRs decreased the mRNA level in Beas2B airway epithelial cells (Figure 1B), indicating that all had an inhibitory effect on gene expression in the reporter context. Other chemokine 3′-UTRs of similar lengths had minimal effects on reporter expression (Supplementary Figure S1), indicating that the 3′-UTR effects were sequence specific.

### Disparity of 3′-UTR effects in the reporter context versus the native context

To evaluate the effects of these chemokine 3′-UTRs in their native context, we developed a reporter-free approach based on a dual-CRISPR system (Figure 1C) that was modified from a previously reported system (16). This system expresses two CRISPR gRNAs that simultaneously target the proximal 3′-UTR (after the stop codon) and distal 3′-UTR. To avoid deleting polyA signal sequences and adjacent sequences, distal gRNAs were designed to recognize sequences upstream of the polyA signal of each 3′-UTR (see ‘Materials and Methods’ section). Analysis of PCR products from amplification of DNA isolated from polyclonal populations of transduced cells showed that the dual-CRISPR system resulted in loss of targeted 3′-UTR DNA in a large proportion of cells (Figure 1D). Larger PCR products were also present and represented either untargeted 3′-UTR DNA or 3′-UTR DNA containing small indels (as confirmed using the Surveyor nuclease assay, data not shown). We then used qRT-PCR to detect the steady state mRNA level and found that targeting the chemokine 3′-UTRs had significant effects on expression of each gene (Figure 1E). For six 3′-UTRs, expression was increased as expected based upon the reporter assays. However, targeting of *CXCL1, CXCL6* and *CXCL8* 3′-UTRs unexpectedly decreased expression. Decreased expression was also seen with independent pairs of gRNAs targeting slightly smaller portions of these 3′-UTR DNAs (Supplementary Figure S2). These results demonstrate that these three 3′-UTR sequences had opposite effects on gene expression in the reporter context and the native context. Since deletion of 3′-UTR DNAs was not 100% efficient, our results may underestimate the magnitude of the effect of deleting 3′-UTR DNAs.

### Targeting *CXCL1, CXCL6* and *CXCL8* 3′-UTRs decreased gene transcription

**Figure 2.**
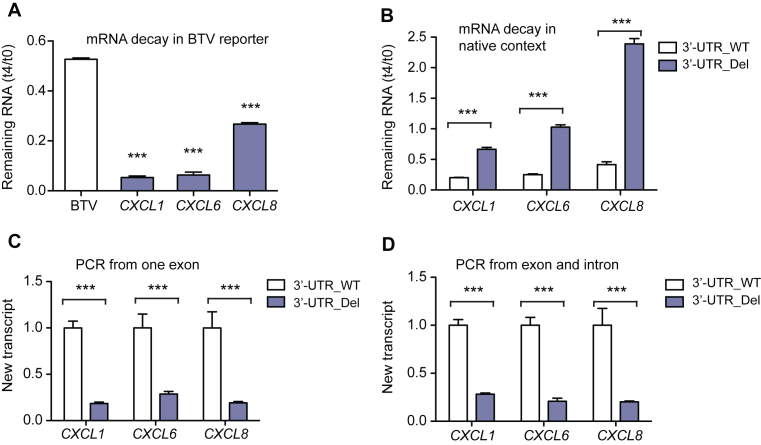
Targeting *CXCL1, CXCL3* and *CXCL8* 3′-UTRs affects both mRNA stability and gene transcription. (**A**) Chemokine 3′-UTR effects on BTV reporter mRNA stability. *GFP* mRNA was measured in untreated cells (t_0_) and 4 h after addition of Dox (t_4_), which blocks reporter transcription. Values represent mean ± s.d., *n* = 3. (**B**) Effects of targeting chemokine 3′-UTRs on mRNA stability in the native context. Cells were incubated with 4sU to label mRNA for 4 h and 4sU-labeled chemokine mRNA was measured 0 (t_0_) and 4 h (t_4_) later, *n* = 5. 4sU-labeled *GAPDH* mRNA was used as a reference; t_4_/t_0_ < 1 indicates that the chemokine mRNA was less stable than *GAPDH* and t_4_/t_0_ > 1 indicates that the chemokine mRNA was more stable than *GAPDH*. (**C** and **D**) Effects of targeting chemokine 3′-UTRs on transcription. New mRNA transcripts labeled during a 20 min 4sU pulse were quantified by quantitative real-time PCR (qRT-PCR) using primers from a single exon (C, detects unspliced and spliced transcripts) or from an exon and an adjacent intron (D, detects unspliced transcripts), *n* = 5. Values represent mean ± s.d. ***P < 0.001 by two-sided Student’s *t*-test.

We next investigated why targeting *CXCL1, CXCL6* and *CXCL8* 3′-UTRs unexpectedly decreased gene expression. Since 3′-UTRs typically regulate gene expression post-transcriptionally (8), we first analyzed the effects of these 3′-UTRs on mRNA stability. In the context of the BTV reporter, we monitored the effects of these 3′-UTRs on mRNA decay rate by measuring the mRNA remaining 4 h after adding Dox to terminate gene transcription. Results showed that each of the three 3′-UTRs destabilized mRNA (Figure 2A). To measure the effect of these 3′-UTRs on mRNA stability in the native context, we performed a 4sU pulse-chase assay (19) to detect mRNA decay in both untreated and 3′-UTR-targeted Beas2B cells. Targeting these three 3′-UTRs increased mRNA stability, indicating that these 3′-UTRs also destabilize mRNA in their native contexts (Figure 2B). We therefore concluded that effects on mRNA stability could not explain why targeting *CXCL1, CXCL6* and *CXCL8* 3′-UTRs decreased gene expression. We next examined the effects of targeting these 3′-UTRs on gene transcription. We measured newly synthesized chemokine transcripts during a 20 min pulse with 4sU (19). Targeting each of these 3′-UTRs substantially decreased levels of newly synthesized chemokine transcripts (Figure 2C and D), indicating reduced gene transcription. Taken together, we demonstrated that targeting *CXCL1, CXCL6* and *CXCL8* 3′-UTRs had dual and opposing effects on transcription and post-transcriptional regulation. The post-transcriptional effect was overcome by the transcriptional effect.

### *CXCL1, CXCL6* and *CXCL8* 3′-UTR DNAs lacked canonical enhancer activity

**Figure 3.**
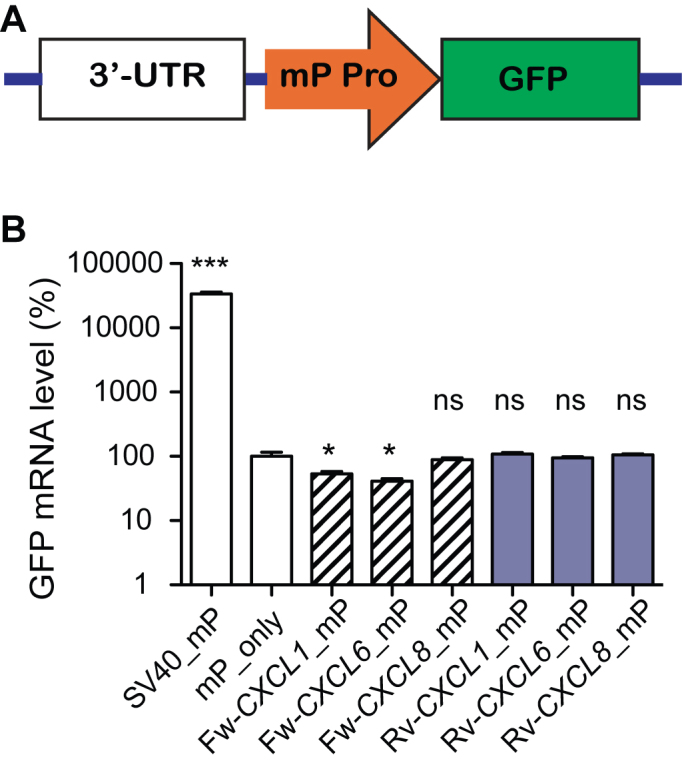
Chemokine 3′-UTR DNA effects on gene expression in an enhancer reporter. (**A**) Diagram of the enhancer reporter. mP Pro is a minimal CMV promoter. (**B**) Chemokine 3′-UTR DNA effects on reporter mRNA. A construct with the minimal promoter but no enhancer sequence (mP_only) served as a negative control; SV40 served as a positive control enhancer. *P < 0.05, ***P < 0.001, n.s., no significant difference by one-way ANOVA and Dunnett’s test, *n* = 5.

To seek further insights, we tested these three 3′-UTR DNAs in a GFP-based enhancer reporter (Figure 3A) (20). Enhancers are generally considered to be orientation independent (23), but orientation-dependent enhancers have been reported (24) so we tested these 3′-UTRs DNAs in both orientations (5′→3′, Fw; 3′→5′, Rv). We transduced Beas2B cells with these reporter viruses and measured reporter mRNA. The expression of GFP mRNA in the whole population of cells was not increased by any of the 3′-UTR DNAs in either orientation (Figure 3B). In contrast, the positive control SV40 enhancer increased mRNA level 300-fold (Figure 3B). We therefore conclude that *CXCL1, CXCL6* and *CXCL8* 3′-UTR DNA sequences lack canonical enhancer activity.

### Modulating gene expression by targeting CRISPR–Cas9 to 3′-UTR *cis*-elements

**Figure 4.**
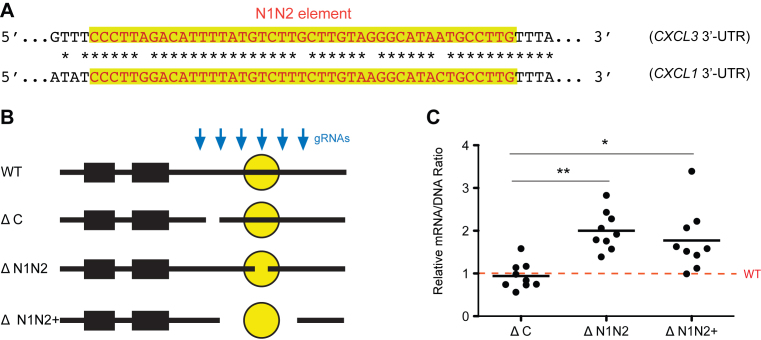
Targeting the N1N2 3′-UTR element in the native context increases *CXCL3* expression. (**A**) The destabilizing element N1N2 (highlighted) in *CXCL3* 3′-UTR and a highly similar sequence from the *CXCL1* 3′-UTR. (**B**) A set of six gRNAs targeting the *CXCL3* 3′-UTR were used to create control deletions that were distant from the N1N2 element (ΔC), short deletions within N1N2 (ΔN1N2) and deletions resulting from a pair of gRNAs that removed N1N2 (ΔN1N2+). (**C**) Effect of *CXCL3* deletions on mRNA level. Each dot represents one of the nine most abundant deletions of the class. *P < 0.05, **P < 0.01 by one-way ANOVA and Dunnett’s test.

Modulating gene expression is critical for understanding biological pathways and could be used therapeutically. 3′-UTRs are a set of readily localized regions that provide a large reservoir of *cis*-regulatory elements that serve as binding sites for RNA binding proteins or microRNAs that decrease or increase mRNA stability and/or translation (7,25). Targeting 3′-UTRs may therefore provide an efficient alternative for modulating gene expression. To test this concept, we used six gRNAs to target a known inhibitory element (N1N2) in the *CXCL3* 3′-UTR (11) and its surrounding regions (Figure 4A). This caused short deletions within or distant from N1N2 (likely directed by single gRNAs) and longer deletions (likely directed by a pair of gRNAs) that excised N1N2 and some surrounding sequence from the genome (Figure 4B). As expected, deletions within N1N2 and longer deletions that removed N1N2 increased *CXCL3* mRNA levels whereas control deletions did not (Figure 4C). This demonstrates that targeting individual 3′-UTR elements can be an alternative approach for modulating gene expression.

### A highly parallel approach for localizing active 3′-UTR elements in their native context

**Figure 5.**
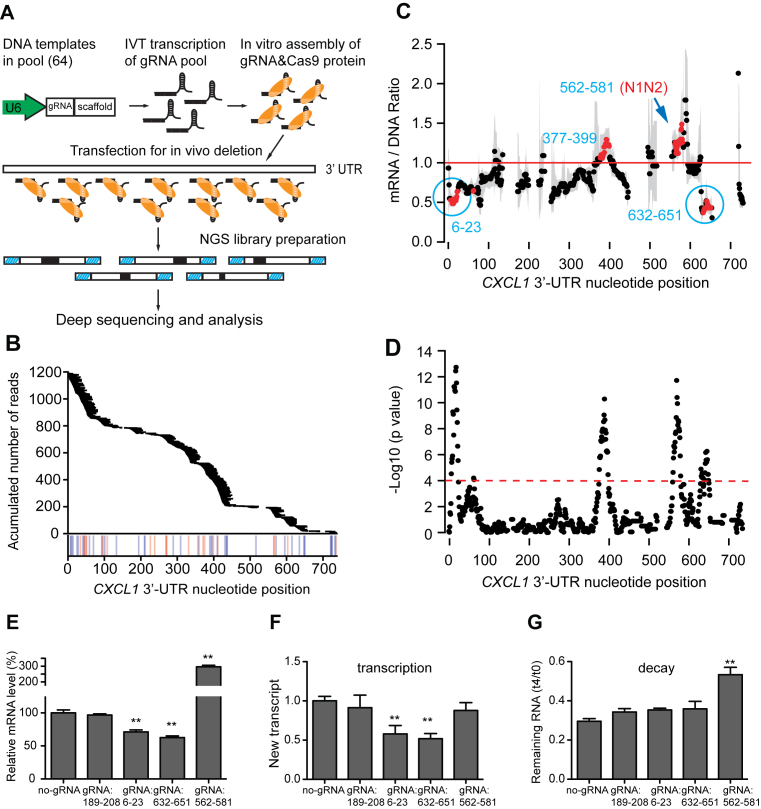
Highly parallel localization of active elements of 3′-UTRs. (**A**) Method for highly parallel analysis of the effects of targeting all possible gRNA sites in the *CXCL1* 3′-UTR. (**B**) *CXCL1* 3′-UTR deletion coverage. Horizontal bars indicate deletions found in DNA and mRNA. Vertical lines indicate the positions of gRNA protospacer adjacent motif (PAM) motifs (NGG/CCN, on plus/minus strand). (**C**) The median normalized mRNA level for all deletions involving a given nucleotide. Gray bars represent 95% confidence intervals. Red dots represent locations with a significant change in mRNA level. (**D**) *P*-values from Mann–Whitney U-test. Dashed line represents Bonferroni-adjusted significance threshold. (**E**) Validation of the effects of selected regions with individual gRNAs on steady state mRNA level. (**F**) Effects of targeting with individual gRNAs on transcription. BEAS2B cells were treated with CRISPR/Cas9 targeting indicated regions. New mRNA transcripts labeled during a 20 min 4sU pulse were quantified by qRT-PCR using primers from a single exon as in Figure 2C. (**G**) Effects of targeting with individual gRNAs on mRNA stability. After targeting with gRNAs, BEAS2B cells were incubated with 4sU for 4 h and *CXCL1* mRNA stability was determined by qRT-PCR analysis of 4sU-labeled mRNA after 0 and 4 h with the same approach used in Figure 2B. Values for E–G represent mean ± s.d., *n* = 5, **P < 0.01 by one-way ANOVA and Dunnett’s test.

3′-UTRs modulate gene expression via *cis*-elements, but experimentally predicting and cataloging these elements remains challenging. Reporter assays can be used to localize *cis*-elements in heterologous contexts but they may not reflect the influence of the native context, so we sought to develop a high-throughput method for localizing 3′-UTR elements in their native context. The *CXCL3* N1N2 targeting experiment demonstrated that the combination of CRISPR/Cas9-guided *in situ* mutations with massively parallel sequencing can be used to map active elements in 3′-UTRs. This inspired us to create a large set of deletions in 3′-UTRs using all possible CRISPR gRNAs that recognize the 3′-UTR DNA. The effects of targeting were determined by measuring mRNA levels using massively parallel sequencing. We applied this strategy to localize regulatory elements in the *CXCL1* 3′-UTR, which is 781 nt-long. We synthesized a DNA oligonucleotide pool containing all 64 possible gRNA sequences targeting the *CXCL1* 3′-UTR. Following *in vitro* transcription, the gRNA pool was combined with Cas9 protein and introduced into BEAS-2B cells by transfection (26), followed by analysis of *CXCL1* 3′-UTR DNA and RNA sequences (Figure 5A). This method resulted in a total of 1176 distinct 3–29 nt-long deletions that were sufficiently frequent for analysis (>6.5 reads/10^7^ reads). These deletions covered most of the *CXCL1* 3′-UTR except for three low GC regions (Figure 5B). Deletions of certain regions, including regions 6–23 and 632–651 led to significant decreases in expression, whereas deletions of other regions, including region 562–581, increased expression (Figure 5C and D). Region 562–581 contains a sequence that is very similar to the N1N2 active element in *CXCL3* and hereafter, we refer to this as the *CXCL1* N1N2 element (Figure 4A).

### Validation of active regions identified using the massively parallel approach

We selected three active regions (6–23, 562–581 and 632–651) and an inactive control region (189–208) for validation with individual gRNAs. As predicted, targeting regions 6–23 and 632–651 decreased *CXCL1* expression, targeting 562–581 (N1N2) increased expression and targeting the negative control region had no significant effect (Figure 5E). To determine whether effects of targeting these regions were due to altered transcription or changes in mRNA stability, we performed 4sU labeling assays after targeting with individual gRNAs (Figure 5F and G). We found that gRNAs that decreased mRNA levels by targeting regions 6–23 and 632–651 decreased transcription with no significant effect on mRNA stability. The magnitude of the effects of targeting these two regions (40 and 45% reduction in transcription, respectively) suggests that these regions explain a substantial portion of the effects of dual CRISPR targeting of almost the entire 3′-UTR (82% reduction, Figure 2C). In contrast, a gRNA that increased the mRNA level by targeting the region 562–581 (N1N2) increased mRNA stability and had no significant effect on transcription. These results validate the ability of the massively parallel approach to detect active elements and identify elements with distinct effects on either transcription or mRNA stability.

## DISCUSSION

One consequence of evolution is that the human genome acquired many noncoding sequences that play crucial roles in controlling development, metabolism, and aging via regulation of gene expression (18). Variants in noncoding regulatory sequences are often associated with the risk of disease (27). It therefore becomes a fundamental goal to completely understand the functions of noncoding sequences. Well-characterized noncoding sequences include promoters, enhancers, noncoding RNAs and 5′- and 3′-UTRs of mRNA. We and others have relied extensively on reporter assays to functionally characterize noncoding sequences including 3′-UTRs (11), 5′-UTRs (28), promoters (29) and enhancers (20). Reporter-based methods are efficient but cannot provide complete insights into the function of regulatory elements in their native context. Cre-Lox mediated recombination has been used to study 3′-UTRs in the native context in mice (30), but this approach is costly and time consuming and is not well suited for use outside of mouse models. CRISPR/Cas9-mediated gene editing has recently become a powerful tool for efficient perturbation of genomic sequences (13), providing a powerful approach for investigating the biological effects of noncoding sequences in the native context. By expressing dual CRISPR gRNAs, we could delete large portions of 3′-UTR DNAs. Metabolic labeling with 4sU allowed us to monitor the dynamics of transcription and mRNA degradation with minimal interference with normal cell growth. This approach led us to find disparities between 3′-UTR effects in the artificial versus the native contexts.

3′-UTRs have well known roles in post-transcriptional gene regulation (8,31). Reporter assays showed that each of the nine chemokine 3′-UTRs we studied decreased mRNA levels and that those effects were due to destabilization of the reporter mRNA. Based on these results, we expected that CRISPR targeting of these 3′-UTRs in their native context would increase mRNA levels. This expected result was seen for six of the nine 3′-UTRs we studied. For the remaining three 3′-UTRs, we used the 4sU pulse-chase approach to show that, despite the decreases in steady state mRNA levels, CRISPR targeting still had the expected effect on mRNA stability. Surprisingly, by directly measuring newly synthesized transcripts we found that targeting *CXCL1, CXCL6* and *CXCL8* 3′-UTRs decreased gene transcription. Promoters and enhancers are usually believed to be the crucial *cis*-elements that control transcription. Our work suggests that 3′-UTRs or 3′-UTR DNAs can also contribute to transcriptional regulation. We considered the possibility that the transcriptional effect of targeting *CXCL1, CXCL6* and *CXCL8* 3′-UTRs might be due to deletion of enhancers contained within these 3′-UTR DNAs. Enhancer–reporter assays showed that these 3′-UTR DNAs did not behave as canonical enhancers. It is also possible that CRISPR targeting of *CXCL1, CXCL6* and *CXCL8* 3′-UTRs disrupted genomic structures or epigenetic markers that are essential for efficient transcription. For example, elements upstream of the polyA signal may play roles in regulating polyadenylation, a process that is linked to transcriptional termination (32). Although our strategy was designed to avoid disrupting polyA signal sequences themselves, it is possible that some effects of CRISPR targeting on transcription may be due to disruption of auxiliary sequences involved in polyadenylation and CRISPR targeting could be a useful method for identifying these auxiliary sequences. Additional work will be required to distinguish these possibilities. However, our work suggests that 3′-UTRs may have distinct roles in simultaneously promoting transcription and mRNA decay, which might be beneficial for establishing a new balance in response to rapid environmental changes. A similar dual role was reported for a promoter sequence that promotes both transcription and mRNA decay (33), suggesting regulatory sequences may play more complicated roles than we have thought.

We also sought to modulate gene expression by targeting CRISPR–Cas9 to 3′-UTR *cis*-regulatory elements. Various other approaches have been used previously to modulate gene expression. Transcription can be modulated using customized DNA-binding proteins (34) or by combining gRNAs with nuclease-deactivated forms of Cas9 (dCas9) to repress expression (CRISPRi) (35) or increase expression (CRISPRa) (36). Post-transcriptional gene silencing has been accomplished using small interfering RNAs and short hairpin RNAs (34). Each of these approaches has many applications but are limited by the need to continue to express exogenous proteins or RNAs that may have nonspecific deleterious effects. Alternatively, durable modulation of gene expression could be accomplished by editing endogenous regulatory elements that control gene expression. Targeting of promoters, enhancers and silencers is feasible but challenging since these regions and the regulatory elements that they contain are often difficult to localize. 3′-UTRs contain hundreds of thousands of predicted *cis*-elements recognized by RNA-binding proteins and miRNAs (6,25) and many more active elements that have been identified experimentally (11). We demonstrated that targeting CRISPR–Cas9 to a known 3′-UTR *cis*-regulatory element, the *CXCL3* N1N2 element, increased *CXCL3* expression 2-fold (Figure 4C). Targeting 3′-UTR elements with individual gRNAs or gRNA pairs allows for various modulating effects on gene expression and is not limited by the requirement of optimizing alternative dCas9 fusion proteins for robust activity found with CRISPRa/i (26). Targeting 3′-UTRs by delivery of *in vitro* transcribed gRNAs and Cas9 protein avoids the continuous expression of exogenous proteins, which can be beneficial for animal model generation and therapeutic applications (37). However, targeting 3′-UTRs can be limited by lack of gRNA targets in AT-rich regions or by lack of active regulatory elements in some genes.

We extended the CRISPR-based 3′-UTR targeting method by using a gRNA library for high-throughput localization of regulatory elements in full length 3′-UTRs. Reporter assays in combination with massively parallel sequencing have previously been used to systematically localize *cis*-elements in regulatory sequences (11,33). CRISPR–Cas9 was recently used for screening of DNA regulatory elements in enhancers and promoters (14,38,39), representing a promising approach that can be used to dissect noncoding regulatory sequences in the native contexts. Similarly, CRISPR–Cas9 can be used for high-throughput dissection of functional enhancer–promoter connections with CRISPRi (40) and for pooled screens for identification of functionally important noncoding regions (41). Libraries contained paired gRNAs have also been used to allow for identification of active DNA regulatory elements by deleting kilobase-long segments of DNA (42). Since enhancer and promoter sequences are not transcribed, enhancer mutations must be inferred from analysis of lentiviral sequences that are transcribed into gRNAs. In contrast, because the 3′-UTR is located within gene transcripts, functional effects can be directly linked to mutations by using massively parallel sequencing to simultaneously detect mutations and transcript levels, which allows for high-throughput localization of active 3′-UTR regulatory elements in the native contexts. Since there is no requirement for inferring mutations from gRNA sequences, we were able to use recombinant Cas9 and *in vitro* transcribed gRNA as an alternative for 3′-UTR targeting, which should make this approach particularly useful in cases cells that are difficult to transduce with lentivirus. Therefore, our reporter-free method for targeting 3′-UTRs is suitable for functional annotation of 3′-UTRs in the native context. We used the high-throughput approach to identify regions that regulate mRNA abundance, and then used individual gRNAs and 4sU labeling to determine how targeting these regions affected transcription and mRNA stability. In principle, it should also be possible to used pooled gRNAs in combination with 4sU labeling to directly identify regions that regulate transcription and stability.

## Supplementary Material

Supplementary Data
