# Supplementary material for: CRISPR–Cas9-mediated functional dissection of 3′-UTRs
Source: Nucleic Acids Res. 2017 Aug 3;45(18):10800–10. doi: 10.1093/nar/gkx675 (PMC5737544; doi:10.1093/nar/gkx675)
Supplement: Supplementary Data [file gkx675_supp.pdf]

Supplementary Figures

CRISPR-Cas9 mediated functional dissection of 3'-UTRs

Wenxue Zhao, David Siegel, Anne Biton, Olivier Le Tonqueze, Noah Zaitlen, Nadav Ahituv & David J Erle

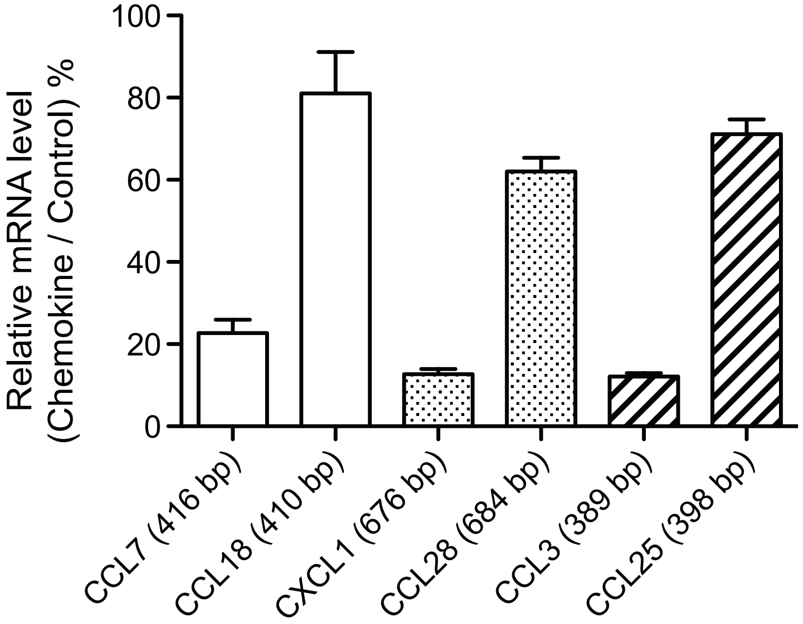

Figure S1: **3'-UTR length is not the primary determinant of functional activity in the reporter assay.** 3'-UTRs were cloned into the BTV reporter and steady state mRNA levels were measured as in Figure 1 A and B. Lengths of each 3' UTR DNA segment are shown in parentheses. Three different fills (blank, dots, and slash) represent different groups of 3'-UTRs with a similar length.

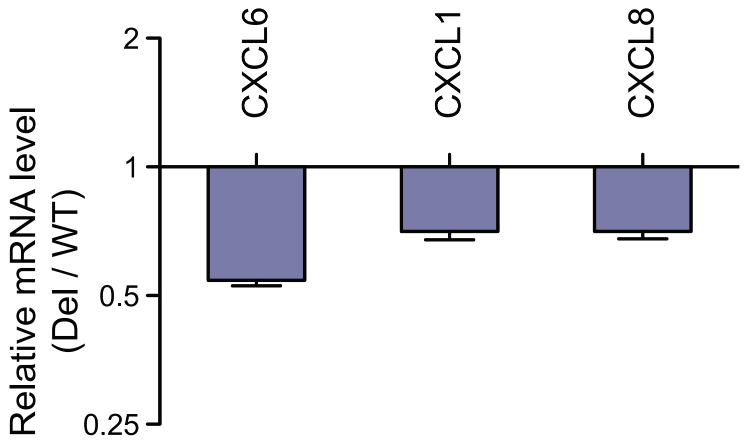

Figure S2. Evaluation of the effects of targeting *CXCL1*, *CXCL6*, and *CXCL8* 3'-UTRs with independent pairs of gRNAs. Experiments were performed as described in Fig. 1E. gRNA positions are provided in Supplementary Data.

Supplementary Data

Oligonucleotide sequences

I. To reconstruct pLX-dual-gRNA and pCW-Csy4-hygromycin (Related to Figure 1B)

pLX-NdeI-F1	TGGACTATCATATGCTTACCG
pLX-R1	AATACCTGCACTGCCTATACGGCAGTGAACGGTGTTTCGTCCTTTCCACA
pLX-F2	TTCACTGCCGTATAGGCAGTGCAGGTATTAATGACCTGCAGTTTTAGAGCTAGAAATAGC
pLX-NheI-R2	AATTGCTAGCTAGAGAGGTACCTCGAGCGG
Csy4-NheI-F	ACTTGCTAGCgccaccATGGACCACTACCTCGACATTCGC
Csy4-BamHI-R	GCGCGGATCCttaGAACCAGGGAACGAAACCTCCTTT
hPGK-SpeI-F	CGCACGTCTCACTAGTACCCT
hPGK-R	GGTGAATTGCTGGGGAGAGAG
Hyg-F	ATCACCGACCTCTCTCCCCAGCAATTCACCATGAAGAAGCCCGAACTCACC
Hyg-XbaI-R	GCGCTCTAGACATTGGACCAGGGTTTTCTTCAACATCACCACAAGTGAGGAGAGAACCTCTACCTTCTTAAACTCGACCTACCTCCTTAG

II. Oligonucleotides for making pLX-dual-gRNAs (Related to Figure 1C-E, S2)

1. The common oligonucleotides

Mid90	TTAGAGCTAGAAATAGCAAGTTAAAATAAGGCTAGTCCGTTATCAACTTGAAAAAGTGGCACCGAGTCGGTGCGTTCACTGCCGTATAGG
Attachment1	ATCAGTGACCTGCTTCATAGGCAG
Attachment2	GACATGTACCTGCGGTGCTAAAAC

2. Dual gRNAs containing oligonucleotides for targeting most of the full length 3'-UTR

Forward primer Name	Forward primer sequences (sgRNA_1)	Reverse primer Name	Reverse primer sequences (sgRNA_2)	Gene symbol	Stop to sgRNA_1*	sgRNA_2 to polyA**	Used in Fig.
sgCK_1_F	GACCTGCTTCATAGGCAGgtgagcttcctcctcccttcGTTTTAGAGCTAGAAATAGCAA	sgCK_1_R	TACCTGCGGTGCTAAAACctttgttctaagccagaaacCTGCCTATACGGCAGTGAACGC	CXCL1	1	116	1D,E
sgCK_2_F	GACCTGCTTCATAGGCAGgggaggaggaagctcactggGTTTTAGAGCTAGAAATAGCAA	sgCK_2_R	TACCTGCGGTGCTAAAACgaaacactgtaaaactaccaCTGCCTATACGGCAGTGAACGC	CXCL1	27	131	S2
sgCK_3_F	GACCTGCTTCATAGGCAGgtatcattgacacttcctgcGTTTTAGAGCTAGAAATAGCAA	sgCK_3_R	TACCTGCGGTGCTAAAACttggaaaagagaatttatcaCTGCCTATACGGCAGTGAACGC	CXCL3	49	62	1D,E
sgCK_5_F	GACCTGCTTCATAGGCAGctgggcaattttatgatgcaGTTTTAGAGCTAGAAATAGCAA	sgCK_5_R	TACCTGCGGTGCTAAAACaatgacaatacaagtaccaaCTGCCTATACGGCAGTGAACGC	CXCL6	14	27	1D,E
sgCK_6_F	GACCTGCTTCATAGGCAGaaactgctccgctgaagactGTTTTAGAGCTAGAAATAGCAA	sgCK_6_R	TACCTGCGGTGCTAAAACaaaaccaacatgacacacagCTGCCTATACGGCAGTGAACGC	CXCL6	32	46	S2
sgCK_9_F	GACCTGCTTCATAGGCAGctggcatcttcactgattctGTTTTAGAGCTAGAAATAGCAA	sgCK_9_R	TACCTGCGGTGCTAAAACaatttttttactgtttctgCTGCCTATACGGCAGTGAACGC	CXCL8	23	81	1D,E
sgCK_10_F	GACCTGCTTCATAGGCAGagatttgcttgaagtttcacGTTTTAGAGCTAGAAATAGCAA	sgCK_10_R	TACCTGCGGTGCTAAAACtacaatcagaaacagtaaaaCTGCCTATACGGCAGTGAACGC	CXCL8	42	52	S2
sgCK_11_F	GACCTGCTTCATAGGCAGgcatcgattttgctcccctcGTTTTAGAGCTAGAAATAGCAA	sgCK_11_R	TACCTGCGGTGCTAAAACtttgatctttcaacatttagCTGCCTATACGGCAGTGAACGC	CXCL10	3	24	1D,E
sgCK_13_F	GACCTGCTTCATAGGCAGtatcaaaacatatgaagtccGTTTTAGAGCTAGAAATAGCAA	sgCK_13_R	TACCTGCGGTGCTAAAACaaattttacagtatataggcCTGCCTATACGGCAGTGAACGC	CXCL11	25	72	1D,E
sgCK_19_F	GACCTGCTTCATAGGCAGtcgctgggcctcgaagcttcGTTTTAGAGCTAGAAATAGCAA	sgCK_19_R	TACCTGCGGTGCTAAAACcaggccgatcacagccctgaCTGCCTATACGGCAGTGAACGC	CCL3	6	29	1D,E
sgCK_21_F	GACCTGCTTCATAGGCAGctcagagacaggaagtcttcGTTTTAGAGCTAGAAATAGCAA	sgCK_21_R	TACCTGCGGTGCTAAAACtgtatttgcaacagcagagaCTGCCTATACGGCAGTGAACGC	CCL4	29	61	1D,E
sgCK_23_F	GACCTGCTTCATAGGCAGattatttgtttctcaagtcaGTTTTAGAGCTAGAAATAGCAA	sgCK_23_R	TACCTGCGGTGCTAAAACaaattttcacataaaatagaCTGCCTATACGGCAGTGAACGC	CCL7	27	35	1D,E

* Distance (in nt) from the end of the stop codon to the sgRNA_1 PAM

** Distance (in nt) from the sgRNA_2 PAM to the start of the polyA sequence

3. Oligonucleotides containing single gRNAs targeting single locations in CXCL1 (Related to Figure 5F-G)

Forward primer Name	Forward primer sequences	Reverse primer Name	Reverse primer sequences	Gene symbol
CXCL1(6-23)-F	GACCTGCTTCATAGGCAGgtgagcttcctcctcccttcGTTTTAGAGCTAGAAATAGCAA	CXCL1(6-23)-R	TACCTGCGGTGCTAAAACgaagggaggaggaagctcacCTGCCTATACGGCAGTGAACGC	CXCL1
CXCL1(632-651)-F	GACCTGCTTCATAGGCAGtggtagttttacagtgtttcGTTTTAGAGCTAGAAATAGCAA	CXCL1(632-651)-R	TACCTGCGGTGCTAAAACgaaacactgtaaaactaccaCTGCCTATACGGCAGTGAACGC	CXCL1
CXCL1(N1N2)-F	GACCTGCTTCATAGGCAGcattttatgtctttcttgtaGTTTTAGAGCTAGAAATAGCAA	CXCL1(N1N2)-R	TACCTGCGGTGCTAAAACGGGCACGGGCAGCTTGCCGGCTGCCTATACGGCAGTGAACGC	CXCL1
CXCL1(ctrl)-F	GACCTGCTTCATAGGCAGttttaggtgtaaaataattaGTTTTAGAGCTAGAAATAGCAA	CXCL1(ctrl)-R	TACCTGCGGTGCTAAAACGGGCACGGGCAGCTTGCCGGCTGCCTATACGGCAGTGAACGC	CXCL1

III. Primers for detection of dual CRISPR gRNA caused double deletions of full length 3'UTRs: (Related to Figure 1D)

Forward primer Name	Forward primer sequences	Reverse primer Name	Reverse primer sequences	Gene symbol
ddect_X1-F2	GGAAACTGCATTCGGAAAAC	ddect_X1-R2	gggccagcccatagtttagt	CXCL1
ddect_X3-F1	GACAGACCTTCCCTGAGTGG	ddect_X3-R1	gcatcggatagtccgttttg	CXCL3
ddect_X6-F1	TGGAATGTTCTGGGTAAACCTT	ddect_X6-R1	cagccagagaagagcagagg	CXCL6
ddect_X7-F2	GAACCCATTGCAACCAAGTC	ddect_X7-R1/2	tttttccccaacaatcctgt	CXCL7
ddect_X8-F2	TCTGGGTTTAAGCTTGTGTCC	ddect_X8-R1/2	caaaatgctaaggggaagca	CXCL8
ddect_X10-F1	TTTCCCCAAGTCTTTCATGG	ddect_X10-R1	catgccaccttcttctccag	CXCL10
ddect_X11-F1	TGTTAAGGGGTCCTTGGTTG	ddect_X11-R1	aaggccgacaaaagaaacaa	CXCL11
ddect_X17-F2	AGCAAGACTCCGTCTCCAAA	ddect_X17-R2	cagaccccatttgaaggaaa	CXCL17

CCL3_det_F1	GTGAGGAGTGGGTCCAGAAA	CCL3_det_R1	aaaagcgctcagtaggagga	CCL3
ddect_CC4-F1	CCAAACCAAAAGAAGCAAGC	ddect_CC4-R1	cggtgcaacagtccctagtt	CCL4
ddect_CC7-F1	AGGCTGATGGGCTAGACAGA	ddect_CC7-R1	acaatgtgagcccaacctct	CCL7
ddect_CC17-F1/2	CCTGTGTGACAGCAGCAACT	ddect_CC17-R1	gaaccctcccagagtgacag	CCL17
ddect_CC25-F1/2	GGCCTCTCTCATTGCTTCTG	ddect_CC25-R1	ccacagccctggaatctaag	CCL25
ddect_CC26-F1	GTGGCAAGGCCAGAAGACTA	ddect_CC26-R1	agaattccgtatcggggtct	CCL26

IV. Primers for detection of chemokine mRNA level with qRT-PCR (Related to Figure 1E)

Forward primer Name	Forward primer sequences	Reverse primer Name	Reverse primer sequences	Gene symbol
CXCL1_F4	CCCAAGAACATCCAAAGTGTG	CXCL1_R4	TGGATTTGTCACTGTTCAGCA	CXCL1
CXCL3_F1	gcagggaattcacctcaaga	CXCL3_R1	ggtgctccccttgttcagta	CXCL3
CXCL6_F1	gcttgagtttcctgccagtc	CXCL6_R1	gggaggctcatagtggtcaa	CXCL6
CXCL7_F3	CAGCAACTCACCCTCACTCA	CXCL7_R3	GTTTGTCCTTTGGTGGAGGA	CXCL7
CXCL8_F4	AAGAAACCACCGGAAGGAAC	CXCL8_R4	AATTTCTGTGTTGGCGCAGT	CXCL8
CXCL10_F3	AGGAACCTCCAGTCTCAGCA	CXCL10_R3	TGATCTCAACACGTGGACAAA	CXCL10
CXCL11_F3	GCAGCAAAGCTGAAGTAGCA	CXCL11_R3	ATGCAAAGACAGCGTCCTCT	CXCL11
CXCL17_F5	AGCGTCACCTCACCTGTTCT	CXCL17_R5	TGTCGGTGCAGCTGTAAGTT	CXCL17
CCL3_F2	gcagcagacagtggtcagtc	CCL3_R2	agcagcaagtgatgcagaga	CCL3
CCL4_F5	ACACAGCTGGGTTCTGAAG	CCL4_R5	CTTCCTCGCGGTGTAAGAAA	CCL4
CCL7_F3	GGCTGAGACCAAACCAGAAA	CCL7_R3	CTGTAGCAGCAGGTAGTTGAAG	CCL7
CCL25_F4	CACACCCAAGGTGTCTTTGA	CCL25_R4	CACACCTTCCTGTGTCTCTTG	CCL25
CCL26_F4	GTGGGAGTGACATATCCAAGAC	CCL26_R4	CCTTGGATGGGTACAGACTTTC	CCL26

V. The primers for detection of chemokine transcription in 4sU labeling experiments with qRT-PCR (Related to Figure 2C,D)

<u>1. PCR from the exon/intron</u>

Forward primer Name	Forward primer sequences	Reverse primer Name	Reverse primer sequences	Gene symbol
X1_E.I-F3	CTCTTCCGCTCCTCTCACAG	X1_E.I-R3	actgactgagcgaggctgtc	CXCL1
X6_E.I-F2	ccagcaacctgccctataaa	X6_E.I-R2	ACTTCCACCTTGGAGCACTG	CXCL6
X8_E.I-F1	CAAGAGCCAGGAAGAAACCA	X8_E.I-R1	ggaaaacgctgtaggtcagaa	CXCL8
b-actin-F-3	cttccagcagatgtggatca	b-actin-R-3	aaagccatgccaatctcatc	beta-actin

<u>2. PCR from exon only</u>

Forward primer Name	Forward primer sequences	Reverse primer Name	Reverse primer sequences	Gene symbol
X1_exon-F2	ACCTCCTCGCCAGCTCTTC	X1_exon-R2	GCTACCAGGAGCAGGAGCA	CXCL1
CXCL6_F1	gcttgagtttcctgccagtc	CXCL6_R1	gggaggctcatagtggtcaa	CXCL6
X8_exon-F2	GAGCACTCCATAAGGCACAA	X8_exon-R2	AGCTGCAGAAATCAGGAAGG	CXCL8
b-actin-F-3	cttccagcagatgtggatca	b-actin-R-3	aaagccatgccaatctcatc	beta-actin

VI. The primers for cloning 3'UTRs in the enhancer reporter pLS-mp (Related to Figure 3)

<u>1. Cloning full length chemokine 3'UTRs in pLS-mP</u>

Primers used for inserting 3’ UTR sequences in a forward orientation

Forward primer Name	Forward primer sequences	Reverse primer Name	Reverse primer sequences	Gene symbol
Eh-Fw_X1_F	ATCGTCTAGAcagaagggaggaggaagctcac	Eh-Fw_X1_R	GTAACCTGCAGGcccctttgttctaagccagaaacac	CXCL1
Eh-Fw_X3_F	ATCGTCTAGAgtatcattgacacttcctgcagggtg	Eh-Fw_X3_R	GTAACCTGCAGGtgataaattctcttttccaagggaaagag	CXCL3
Eh-Fw_X6_F	ATCGTCTAGAccatgcatcataaaattgcccagtct	Eh-Fw_X6_R	GTAACCTGCAGGctccaaatgacaatacaagtaccaaaaacc	CXCL6
Eh-Fw_X8_F	ATCGTCTAGAccaagaatcagtgaagatgccagtga	Eh-Fw_X8_R	GTAACCTGCAGGccatacaatcagaaacagtaaaaaaaatttgg	CXCL8
Eh-Fw_X10_F	ATCGTCTAGAAACCAGAGGGGAGCAAAATCGATGC	Eh-Fw_X10_R	GTAACCTGCAGGAACCTTTTGATCTTTCAACATTTAGATAGT	CXCL10
Eh-Fw_C3_F	ATCGTCTAGATTCGAGGCCCAGCGACCTC	Eh-Fw_C3_R	GTAACCTGCAGGACAGCCCTGAACAAAAGCATCCGAT	CCL3
Eh-Fw_C4_F	ATCGTCTAGACAGGAAGTCTTCAGGGAAGGTCAC	Eh-Fw_C4_R	GTAACCTGCAGGTGAAAACACACAGAATCAAATGTGTTATCC	CCL4
Eh-Fw_C7_F	ATCGTCTAGAGACTGAACTGAAAACAAGCCATGAC	Eh-Fw_C7_R	GTAACCTGCAGGTGAAAATTTGGGAGTCATACATATGCAAAT	CCL7

Pmers used for inserting 3’ UTR sequences in a reverse orientation

Forward primer Name	Forward primer sequences	Reverse primer Name	Reverse primer sequences	Gene symbol
Eh-Rv_X1_F	GTAACCTGCAGGcagaagggaggaggaagctcac	Eh-Rv_X1_R	ATCGTCTAGAcccctttgttctaagccagaaacac	CXCL1
Eh-Rv_X3_F	GTAACCTGCAGGgtatcattgacacttcctgcagggtg	Eh-Rv_X3_R	ATCGTCTAGAtgataaattctcttttccaagggaaagag	CXCL3
Eh-Rv_X6_F	GTAACCTGCAGGccatgcatcataaaattgcccagtct	Eh-Rv_X6_R	ATCGTCTAGActccaaatgacaatacaagtaccaaaaacc	CXCL6
Eh-Rv_X8_F	GTAACCTGCAGGccaagaatcagtgaagatgccagtga	Eh-Rv_X8_R	ATCGTCTAGAccatacaatcagaaacagtaaaaaaaatttgg	CXCL8
Eh-Rv_X10_F	GTAACCTGCAGGAACCAGAGGGGAGCAAAATCGATGC	Eh-Rv_X10_R	ATCGTCTAGAAACCTTTTGATCTTTCAACATTTAGATAGT	CXCL10

Eh-Rv_C3_F	GTAACCTGCAGGTTCGAGGCCCAGCGACCTC	Eh-Rv_C3_R	ATCGTCTAGAACAGCCCTGAACAAAAGCATCCGAT	CCL3
Eh-Rv_C4_F	GTAACCTGCAGGCAGGAAGTCTTCAGGGAAGGTCAC	Eh-Rv_C4_R	ATCGTCTAGATGAAAACACACAGAATCAAATGTGTTATCC	CCL4
Eh-Rv_C7_F	GTAACCTGCAGGGACTGAACTGAAAACAAGCCATGAC	Eh-Rv_C7_R	ATCGTCTAGATGAAAATTTGGGAGTCATACATATGCAAAT	CCL7

VII. The oligonucleotide pool for expressing sgRNAs targeting CXCL1 3'UTR (Related to Figure 5A, B)

1. Synthesis of oligo pool: Each oligonucleotide consists of two commone regions and a specific gRNA sequence (XXXXXXXXXXXXXXXXXXXX) as shown below:

TATGCCTGTTGCTGAGCTTGGAAATTAATACGACTCACTATAXXXXXXXXXXXXXXXXXXXXXGTTTTAGAGCTAGAAATAGCAAGTTAAAATAAGGCTAGTCCGTTATCAACTTGAAAAAGTG

Serial_ID	sgRNA sequence contained (XXXXXXXXXXXXXXXXXXXX) *	Modification of gRNA
CXCL1.IVT.sgRNA_1	ggtgagcttcctcctcccttc	+G
CXCL1.IVT.sgRNA_2	ggaagggaggaggaagctcac	+G
CXCL1.IVT.sgRNA_3	gggaggaggaagctcactgg	NA
CXCL1.IVT.sgRNA_4	ggataagggcagggcctccttc	+2G
CXCL1.IVT.sgRNA_5	ggtcactggtggctgttcctga	+2G
CXCL1.IVT.sgRNA_6	ggtggctgttcctgaagg	-2G
CXCL1.IVT.sgRNA_7	ggcttctgttcctataagggca	+2G
CXCL1.IVT.sgRNA_8	ggtcttctgttcctataagggc	+2G
CXCL1.IVT.sgRNA_9	ggttcctcttctgttcctataa	+2G
CXCL1.IVT.sgRNA_10	ggtttcctcttctgttcctata	+2G
CXCL1.IVT.sgRNA_11	ggaaggaggccctgcccttat	+G
CXCL1.IVT.sgRNA_12	ggcccttataggaacagaag	re1G
CXCL1.IVT.sgRNA_13	ggaaagagagacacagctgcag	+2G
CXCL1.IVT.sgRNA_14	ggcacattaggcacaatccagg	+2G
CXCL1.IVT.sgRNA_15	ggaaacacattaggcacaatcc	+2G
CXCL1.IVT.sgRNA_16	ggacacagctgcagaggccacc	+2G
CXCL1.IVT.sgRNA_17	ggaagcgatgctcaaacacatt	+2G
CXCL1.IVT.sgRNA_18	ggaatgtgtttgagcatcgctt	+2G
CXCL1.IVT.sgRNA_19	ggattctatgttaatatttt	re1G
CXCL1.IVT.sgRNA_20	ggttttaggtgtaaaataatta	+2G
CXCL1.IVT.sgRNA_21	ggtttaggtgtaaaataattaa	+2G
CXCL1.IVT.sgRNA_22	ggaatataataggacagtgtgc	+2G
CXCL1.IVT.sgRNA_23	ggtcaaaaagaatgaatataat	+2G
CXCL1.IVT.sgRNA_24	ggatccagattgaactaacttg	+2G
CXCL1.IVT.sgRNA_25	ggaatccagattgaactaactt	+2G
CXCL1.IVT.sgRNA_26	ggaatccagattgaactaact	+G
CXCL1.IVT.sgRNA_27	ggaaccccaagttagttcaatc	+2G
CXCL1.IVT.sgRNA_28	ggattcatatttaatttga	-G
CXCL1.IVT.sgRNA_29	ggaaatattaacataatgac	re1G
CXCL1.IVT.sgRNA_30	ggtcattatgttaatatttctg	+2G
CXCL1.IVT.sgRNA_31	ggcacagtggctggcatgttgc	+2G
CXCL1.IVT.sgRNA_32	ggccagcctctatcacagtggc	+2G
CXCL1.IVT.sgRNA_33	ggtccgccagcctctatcacag	+2G
CXCL1.IVT.sgRNA_34	ggcatgccagccactgtgatag	+2G
CXCL1.IVT.sgRNA_35	ggccagccactgtgatagaggc	+2G
CXCL1.IVT.sgRNA_36	ggccactgtgatagaggctgg	+G
CXCL1.IVT.sgRNA_37	ggatctcattggccatttgct	+G
CXCL1.IVT.sgRNA_38	ggctggcggatccaagcaaa	NA
CXCL1.IVT.sgRNA_39	ggccttcacaatgatctcat	re1G
CXCL1.IVT.sgRNA_40	ggccaatgagatcattgtga	NA
CXCL1.IVT.sgRNA_41	ggaatgagatcattgtgaaggc	+2G
CXCL1.IVT.sgRNA_42	ggatgagatcattgtgaaggca	+2G
CXCL1.IVT.sgRNA_43	ggagatcattgtgaaggcag	re1G
CXCL1.IVT.sgRNA_44	ggaaatgatttcacagtgtg	re1G
CXCL1.IVT.sgRNA_45	ggaaagacataaaatgtccaa	+G
CXCL1.IVT.sgRNA_46	ggaaagacataaaatgtcca	re1G
CXCL1.IVT.sgRNA_47	ggaaaatgttctaaatatccct	+2G
CXCL1.IVT.sgRNA_48	ggcattttatgtctttcttgta	+2G
CXCL1.IVT.sgRNA_49	ggtaaaactaccattaaaca	re1G
CXCL1.IVT.sgRNA_50	ggcatactgccttgtttaa	-G

CXCL1.IVT.sgRNA_51	ggtagttttacagtgtttc	-G
CXCL1.IVT.sgRNA_52	ggtgtttctggcttagaacaa	+G
CXCL1.IVT.sgRNA_53	ggtttctggcttagaacaaa	re1G
CXCL1.IVT.sgRNA_54	ggtttctggcttagaacaaag	+G
CXCL1.IVT.sgRNA_55	ggaaaaaactcgtttgatttt	+G
CXCL1.IVT.sgRNA_56	ggaaaaaactcgtttgattttt	+2G
CXCL1.IVT.sgRNA_57	ggaaaaactcgtttgatttttg	+2G
CXCL1.IVT.sgRNA_58	ggaaaactcgtttgatttttgg	+2G
CXCL1.IVT.sgRNA_59	ggaaactcgtttgatttttggg	+2G
CXCL1.IVT.sgRNA_60	ggttgatttttgggggaaaca	+2G
CXCL1.IVT.sgRNA_61	ggatttttgggggaaacaa	re1G
CXCL1.IVT.sgRNA_62	ggatcaccagattttccagtaa	+2G
CXCL1.IVT.sgRNA_63	ggaaacaagggctacctttac	+G
CXCL1.IVT.sgRNA_64	ggctacctttactggaaaatc	+G

* Note: gRNAs were modified to begin with two Gs as needed. +G, one additional G was added at the 5' end;
+2G, two additional Gs was added at the 5' end;
re1G, the firest nucleotide at the 5' end was replaced with G;
-G, the first nucleotide at the 5' end was removed;
NA, no modification was made with the gRNA.

2. Primers for PCR amplification of the oligonucleotide pool: **(Related to Figure 5)**

Forward primer Name	Forward primer sequences	Reverse primer Name	Reverse primer sequences
IVT.lib_CXCL1	tatgcctgttgctgagcttg	IVT.lib_R	AAAAAAAGCACCGACTCGGTGCCACTTTTTCAAGTTGATAACGGACTAGC
CXCL1.P1_F20	acagtgacaaatccaactga	CXCL1.P3_R20	accagattttccagtaaagg
CXCL1full_Nx_F	cctactgggaagaagttgagagtgaggatgggtccaatggacagtgacaaatccaactga	CXCL1full_Nx_R	gacagactcgttgacagctgtacgtaacgctactcaggcaaccagattttccagtaaagg

VIII. sgRNA sequences targeting CXCL3 N1N2 and PCR primers for sequencing library (Related to Figure 4)

sgRNA sequences targeting CXCL3 3'-UTR DNA

CXCL3_outside N1N2	ATTGAAATGCAAGCAATTAG
CXCL3_outside N1N2	GCAATTAGTGGATCACTGTT
CXCL3_outside N1N2	AGTGGATCACTGTTAGGGTA
CXCL3_outside N1N2	TTCTGCAGCGTTTCTCTTTC
CXCL3_outside N1N2	TGCAGCGTTTCTCTTTCCCT
CXCL3_inside N1N2	TTAGACATTTTATGTCTTGC

For Fisrt Round PCR:

fX3crNv-F1	ACACTCTTTCCCTACACGACGCTCTTCCGATCTNNNNATGACAGGGTGGGGAACTGGAGGG
fX3crNv-F2	ACACTCTTTCCCTACACGACGCTCTTCCGATCTNNNNGTGTTCAACATTTTTATGCTGAAG
fX3crNv-R	GTGACTGGAGTTCAGACGTGTGCTCTTCCGATCNNNNgtacaaatgtaacagtAATGATAAATTCTC

For Second Round PCR:

D2nd-F	AATGATACGGCGACCACCGAGATCTACACNNNNNNNNACACTCTTTCCCTACACGACG
D2nd-R	CAAGCAGAAGACGGCATACGAGATNNNNNNNNGTGACTGGAGTTCAGACGTGT
